# The identification of *PAX7* variants and a potential role of muscle development dysfunction in congenital scoliosis

**DOI:** 10.1186/s13619-022-00116-9

**Published:** 2022-05-02

**Authors:** Muchuan Wang, Ziquan Li, Sen Zhao, Zhifa Zheng, Yipeng Wang, Guixing Qiu, Zhihong Wu, Nan Wu, Terry Jianguo Zhang, Siyi Cai

**Affiliations:** 1 Department of Orthopedic Surgery, State Key Laboratory of Complex Severe and Rare Diseases, Peking Union Medical College Hospital, Peking Union Medical College and Chinese Academy of Medical Sciences, Beijing, 100730 China; 2 Beijing Key Laboratory for Genetic Research of Skeletal Deformity, Beijing, 100730 China; 3 Key laboratory of big data for spinal deformities, Chinese Academy of Medical Sciences, Beijing, 100730 China; 4 Medical Research Center, Peking Union Medical College Hospital, Peking Union Medical College and Chinese Academy of Medical Sciences, No. 1 Shuaifuyuan, Beijing, 100730 China; 5 Department of Molecular and Human Genetics, Baylor College of Medicine, Houston, TX 77030 USA

Dear Editor,

Congenital scoliosis (CS) is a spinal malformation characterized by failure of vertebral formation or segmentation, or a mix of these deformities, resulting in longitudinal and rotational imbalance, and affects 0.05-0.1% of newborns (Wu et al. 2015). It is generally understood that the development of CS has an underlying genetic basis. Specifically, genes related to somite regulation or osteogenesis during embryonic development are believed to be responsible for the vertebral malformations observed in CS patients (Pourquié 2011).

*PAX7* is a pioneer transcription factor that controls cell fate specification through chromatin remodeling. It is critical to regulate satellite cell expansion and differentiation during both neonatal and adult myogenesis (Mayran et al. 2018). Bi-allelic variants in *PAX7* have been shown to cause progressive congenital myopathy with scoliosis (MYOSCO). Patients develop muscle weakness and atrophy in the early postnatal period and deteriorate with age (Feichtinger et al. 2019). Previous studies have reported the impact of *PAX7* loss-of-function in human muscle development and related diseases. However, the role of *PAX7* variants in congenital vertebral malformations, particularly CS, remains understudied.

Considering that scoliosis is within the phenotypic spectrum of the *PAX7*-related myopathy described above, gene variants of *PAX7* are likely to play an essential role in isolated CS. Understanding this relationship has the potential to improve the diagnostic rate of such diseases and further expand our understanding of how muscle development influences vertebral development.

We identified nine individuals with CS who carried rare variants of *PAX7* from a Chinese cohort of vertebral malformations in the Deciphering disorders Involving Scoliosis and COmorbidities (DISCO) study (http://discostudy.org/) at Peking Union Medical College Hospital (PUMCH) (total cohort size *n* = 583). Exome sequencing and bioinformatics data analyses were performed, and variants in genes associated with CS were not detected (materials and methods are shown in supplementary material). We identified six different disease-causing candidate variants in the *PAX7* gene (NM_002584.2) based on each variant’s predicted deleterious functional impact, including 5 heterozygous missense variants and a heterozygous stop-gain variant locating on exons 6 and 8 of *PAX7* isoforms 1 and 2 (Table 1). All variants were previously unreported in the public database. CADD scores of the missense variants ranged from 7.196 to 15.951 and are 37 for the stop-gained variant. Furthermore, the missense variant c.917C > T, p.Pro306Leu is predicted to be pathogenic by SIFT and Variant Assessor.

**Table 1:** Genetic description of the *PAX7* variants and clinical features of the patients

	Individual 1	Individual 2	Individual 3	Individual 4	Individual 5	Individual 6	Individual 7	Individual 8	Individual 9
Patient ID	SCO2003P0422	SCO2003P0034	SCO2003P0217	SCO2003P0227	SCO2003P0149	SCO2003P1819	SCO2003P1851	SCO2003P1969	SCO2003P2362
Age	13	14	11	10	12	15	13	3	7
Gender	Female	Female	Female	Female	Female	Female	Male	Female	Male
Mutation Type	Missense	Missense	Missense	Missense	Stop-gained	Missense	Missense	Missense	Missense
cDNA Variant (NM_002584.2)	c.845C > T	c.1375G > A	c.1376G > A	c.917C > T	c.1485C > A	c.1360G > A	c.1387G > A	c.845C > T	c.1387G > A
Protien Variant	p.Ala282Val	p.Gly459Ser	p.Gly459Asp	p.Pro306Leu	p.Tyr495Ter	p.Val454Met	p.Gly463Ser	p.Ala282Val	p.Gly463Ser
gnom AD Frequency	6.47E-05	1.00E-03	0	0	0	0	9.69E-05	6.47E-05	9.69E-05
CADD score	14.8	11.96	14.01	15.95	37	7.196	10.38	14.8	10.38
GERP++ score	4.82	2.94	3.91	3.75	−1.68	4.84	2.93	4.82	2.93
SIFT	Tolerated	Tolerated low confidance	Deleterous low confidance	Deleterous	N/A	Tolerated low confidance	Tolerated low confidance	Tolerated	Tolerated low confidance
Mutation Assessor	Low	N/A	Low	Medium	N/A	N/A	N/A	Low	N/A
CS Classification	III	II	III	III	II	III	I	I	III
Vertebral Abnormalities	Vertebral fusion (L1-2); Spine bifida (L2, L3, L5, S2)	Failure of segmentation (T7-9)	Failure of segmentation (T1-T7); Butterfly vertebra (T6); Absence of T2 vertebra	Vertebral fusion (C7-T10); Butterfly vertebra; Hemivertebra	Lamina fusion (T5-9); Failure of segmentation	Failure of segmentation (C3-5); Hemivertebra (T3)	Butterfly vertebra (L4); Hemivertebra (L5)	Butterfly vertebra (T2, T3); Hemivertebra (T5, T7)	Failure of segmentation (C3-5); Spine bifida (T1); Cuneiform vertebrae (T6)
Skeletal deformity	No	Pigeon chest; Rib fusion (5,6th)	No	No	No	No	Sacrum deformity	Absence of ribs (5,6th)	No
Craniofacial	Normal	Normal	Normal	Normal	Normal	Normal	Normal	Normal	Normal
Respiration	Normal	Normal	Restrictive ventilation dysfunction	Restrictive ventilation dysfunction	Restrictive ventilation dysfunction	Normal	Normal	Normal	Congenital atelectasis of left lung
Muscle tonus	Normal	Normal	Normal	Normal	Normal	Normal	Normal	Normal	Upper limbs grade 4; Lower limbs grade 4+
Cardiac	Normal	Normal	Tricuspid regurgitation (mild-moderate)	Normal	Normal	Normal	Normal	Normal	Patent ductus arteriosus
Spinal cord	Diaste-matomyelia; Low position spinal cord; Sacral canal cyst	No	Syringomyelia	Diaste-matomyelia; Syringo-myelia	Diaste-matomyelia	No	No	No	Syringomyelia (T4-10)
Other deformities	None	None	None	Absence of spleen	None	None	None	None	None

*cDNA* complementary DNA

The clinical data for each affected individual with a *PAX7* variant is summarized in Table 1. All individuals from our cohort had uneventful prenatal histories. The earliest diagnostic age of CS was 3 years old, while the mean age was 10.9 ± 3.8. Two patients presented with only vertebral malformations (including butterfly vertebrae and hemivertebrae) were categorized to have Type I CS. Two patients who presented with only failure of segmentation (whether vertebral or laminar fusion) were categorized as Type II CS. The remaining five patients were diagnosed with Type III CS, suffering both types of spinal deformities (radiological data is summarized in Figure 2 in supplementary material). The most frequent location of vertebral malformation was the thoracic spine, followed by the lumbar and cervical spine. Failure of segmentation led to rib fusion and pigeon chest in one patient, while hemivertebrae of thoracic and lower lumbar spine resulted in the absence of ribs and sacrum deformity, respectively, in two patients. Comorbidities included restrictive ventilation dysfunction due to decreased thoracic volume led by severe scoliosis, endured by three patients; one patient had congenital atelectasis of the left lung; two patients had congenital heart disease (mild-moderate tricuspid regurgitation in one patient and patent ductus arteriosus in the other). Intraspinal anomalies, including diastematomyelia and syringomyelia, were found in five individuals through spinal MRI. Interestingly, according to the British Medical Research Council scale, only one patient showed mild muscle tonus decrease (grade 4/5 in upper limbs and grade 4+/5 in lower limbs).

This study provides evidence that heterozygous missense and stop-gain variants in the *PAX7* gene may be a novel genetic cause of CS or congenital vertebral malformation. These variants have high pathogenicity prediction scores and are previously unreported.

In previously published reports, phenotypes associated with *PAX7* mutations varied from cleft lip to hypotonia, muscle weakness, MYOSCO, and postnatal growth retardation (Summarized in Table 2 in supplementary material). The predominant phenotype of our patients was congenital scoliosis, characterized by either failure of vertebral formation, segmentation, or mixed deformity, expanding the phenotypic spectrum of *PAX7*-related diseases. Intraspinal anomalies including diastematomyelia and syringomyelia were found in 5 individuals through spinal MRI. There was no evidence of significant muscle tonus decrease or craniofacial malformation in our cohort. Possible mechanisms underlying the phenotypic differences between our group of patients and those described previously include 1) variants in previous studies are mainly located near or within the paired box domain, which is highly conserved across species; 2) during early stages of embryo development, PAX7 expression in neural crest cells (NCCs) displays a migratory pattern. Therefore its functions in the formation of cartilage and bone of the craniofacial skeleton and neural system development may result from the variation of phenotypes from cleft lip/palate to intraspinal anomalies; 3) different variants may affect the level of PAX7 expression to different extents. The PAX7 variants in our cohort were generally localized on exon 6 and 8, surrounding the paired box protein domain (Shown in Figure 1 in supplementary material), while the phenotypes varied. There may not be a strong correlation between PAX7 variants and CS classification since the patients sharing the same mutation sites or location within exons presented differently in vertebral abnormities regarding formation or segmentation failure. Further, we did not characterize the associations between distinct PAX7 gene variants and specific sub-phenotypes, including the location of vertebral anomalies, intraspinal anomalies, and extra-spinal skeletal malformations.

Paraspinal muscle plays an important role in the posture and movement of the spine. Asymmetry of the paraspinal musculature and imbalance of its strengths are likely to result in scoliosis. Differences in transcriptional profiling or dysregulated expression of genes related to the TGF-β pathway between the concave and convex side of the spinal curve were previously identified in the paravertebral muscles of adolescent idiopathic scoliosis (AIS) patients (Buchan et al. 2014). Scoliosis is a common manifestation in myopathy diseases, including Duchenne muscular dystrophy (DMD) and dystonia diseases such as Parkinson’s Disease (PD). Scoliosis involving vertebral malformations has been found in patients with variants in genes related to muscle function or myogenesis but with low frequency. Zieba et al. identified an autosomal dominant form of spondylocarpotarsal synostosis (SCT) due to heterozygous missense or nonsense variants in *MYH3* (Zieba et al. 2017). These patients had comorbidities of progressive scoliosis ranging from mild disc space narrowing to severe vertebral fusion. *MYH3* encodes embryonic myosin heavy chain 3 and functions in muscle contraction by binding to and exerting force on actin filaments through the hydrolysis of ATP. In wild-type mice, its expression can be seen postnatally in the bone and muscle between the neural arches of the cervical and thoracic spine. *MYH3* variants can also cause CS in Freeman-Sheldon Syndrome. More recently, biallelic homozygous loss-of-function variants in *MYF5*, a key regulator for myogenesis’s early stage, were found to be associated with congenital external ophthalmoplegia, vertebral, and rib anomalies in three families (Di Gioia et al. 2018). Additionally, researchers reported kyphoscoliosis in 6 of 15 pediatric-onset cardiomyopathy patients with variants of *ALPK3.* These variants were predicted to have “muscle contraction” and “myogenesis” phenotypes by GeneNetwork Assisted Diagnostic Optimization (Herkert et al. 2020). Previous studies of NCC demonstrated that PAX7 staining occurs throughout the presumptive musculature surrounding the ribs and vertebrae in Carnegie Stage 18 embryos, which may interfere with the process of segmentation or vertebrae development (Betters et al. 2010). It is speculated that muscle function or myogenesis-related genes may affect vertebrae segmentation and spine growth by affecting the forces surrounding the intervertebral disc. Indeed, the paraspinal muscles, especially small multifidus muscles, connecting to the neural arches in early life stages have been implicated in causing impropriate vertebral segmentation or formation. Axial hypotonia may accelerate the progression of scoliosis with the growth of the patient’s age.

In conclusion, we find that heterozygous pathogenic variants in *PAX7* are associated with CS without notable dysfunction in the appendicular skeleton muscle. These findings provide important implications for diagnosis and a better understanding of the etiology of CS.

## Supplementary Information

**Additional file 1: Figure 1.** Distribution of variant on PAX7 protein. **Figure 2.** Radiological characteristics of the CS patients carrying PAX7 variants. **Table 2.** PAX7 mutations and their associated phenotypes in previously published reports.

## Data Availability

The datasets used and/or analyzed during the current study are available from the corresponding authors upon reasonable request.

## Abbreviations

CS: Congenital scoliosis
DISCO: Deciphering disorders Involving Scoliosis and COmorbidities
PUMCH: Peking Union Medical College Hospital
MYOSCO: Myopathy, congenital, progressive, with scoliosis
ES: Exome sequencing
3D: 3-dimensional
CT: Computed tomography
MRI: Magnetic resonance imaging
NCC: Neural crest cell
AIS: Adolescent idiopathic scoliosis
DMD: Duchenne muscular dystrophy
PD: Parkinson’s Disease
SCT: Spondylocarpotarsal synostosis
