# Supplementary material for: The identification of PAX7 variants and a potential role of muscle development dysfunction in congenital scoliosis
Source: Cell Regen. 2022 May 2;11:16. doi: 10.1186/s13619-022-00116-9 (PMC9061922; doi:10.1186/s13619-022-00116-9)
Supplement: Supplementary file 1 — Additional file 1: Figure 1. Distribution of variant on PAX7 protein. Figure 2. Radiological characteristics of the CS patients carrying PAX7 variants. Table 2. PAX7 mutations and their associated phenotypes in previously published reports. [file 13619_2022_116_MOESM1_ESM.docx]

**The identification of *PAX7* variants and a potential role of muscle development dysfunction in congenital scoliosis**

**Supplementary Material**

**Methods**

***Participant recruitment***

Patients with rare variants of *PAX7* were selected from a Chinese cohort of vertebral malformations in the Deciphering disorders Involving Scoliosis and COmorbidities (DISCO) study (<http://discostudy.org/>) at Peking Union Medical College Hospital (PUMCH) (total cohort size n = 583). Exome sequencing (ES) was performed as previously described (Zhao et al., 2021). All patients were diagnosed by spine surgeons during a thorough clinical examination. Image evaluations, including standing full-spine posterior-anterior and lateral radiographs, 3-dimensional (3D) computed tomography (CT) and whole-spine magnetic resonance imaging (MRI) were required. Our study was approved by the ethics committee of PUMCH.

***In-house genomic databases***

An in-house database consisting of ES data from 4246 unrelated Chinese individuals without apparent scoliosis was utilized as a reference to determine the frequency of candidate variants in the Chinese Han population. The Swedish population reference database consists of whole genomes of 4000 individuals.

**Figure 1. Distribution of variant on PAX7 protein.**


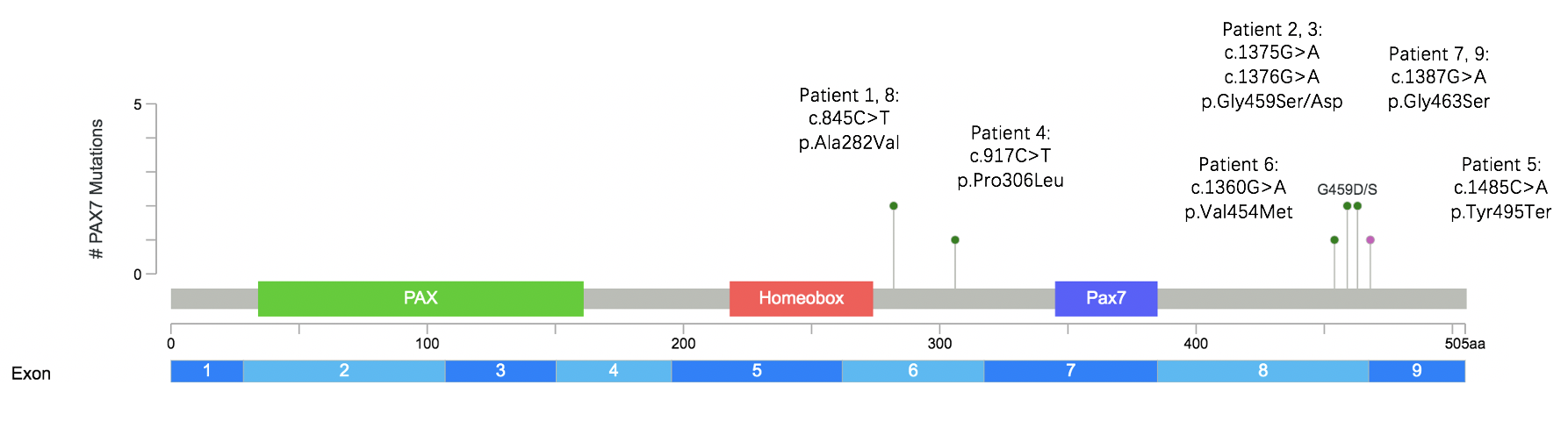


Six identified heterozygous variants in the *PAX7* gene and its protein annotations are shown in green and pink dots, representing missense and stop-gained variants respectively. Green bar, “Paired box” domain (34 - 161); red, Homeobox domain (218 - 274); purple, Paired box protein (345 - 385).

**Figure 2. Radiological characteristics of the CS patients carrying *PAX7* variants**


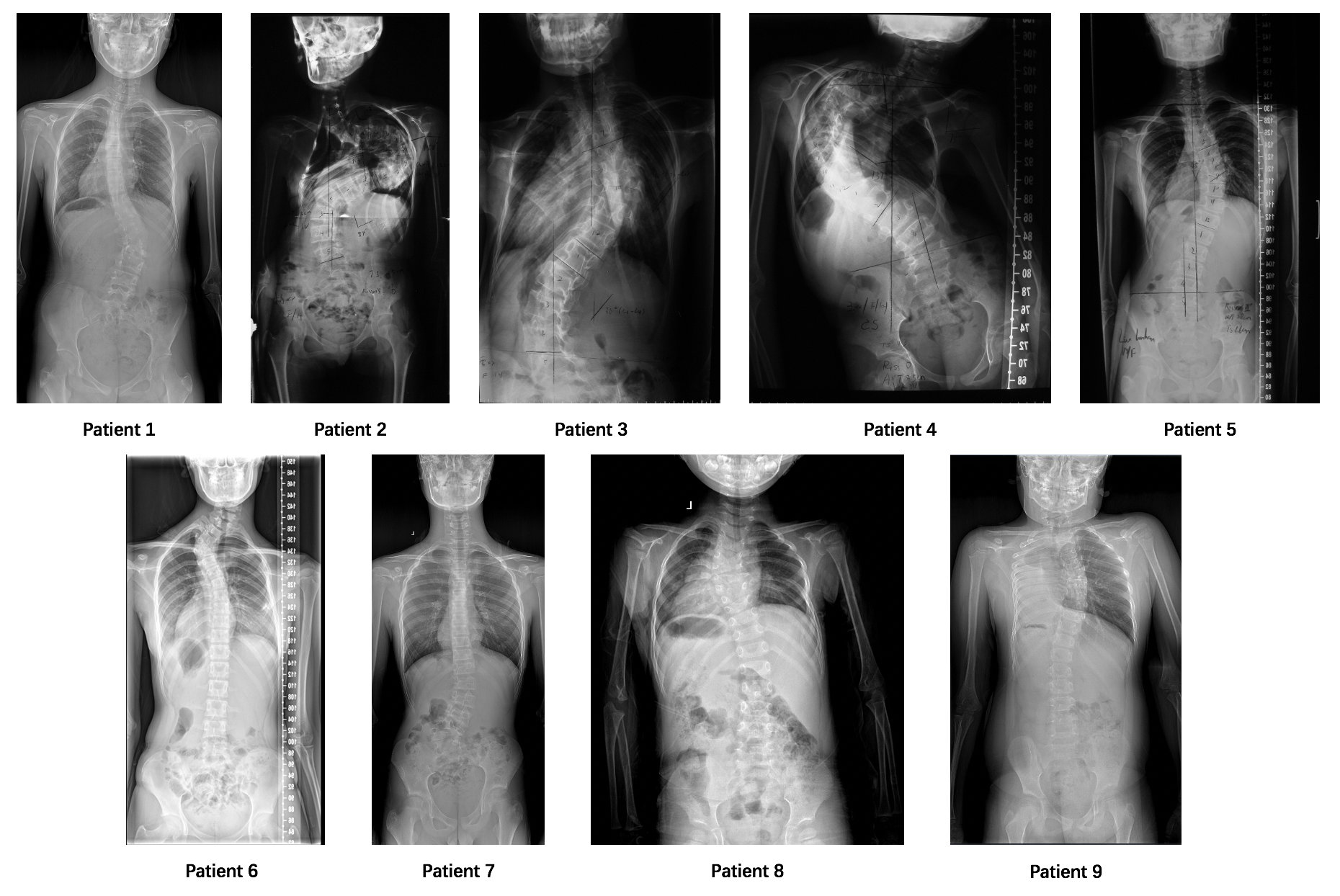


**Table 2 *PAX7* mutations and their associated phenotypes in previously published reports**

|  | Mutation Type | cDNA Variant （NM_002584.2） | Protien Variant | Zygosity | Exon | Domain | Reported Phenotype |
| --- | --- | --- | --- | --- | --- | --- | --- |
| Butali et al. 2013 | Missense | c.7G>A | p.Ala3Thr | Heterozygous | 1 |  | Cleft lip with or without cleft palate |
|  | Missense | c.43G>A | p.Ala15Thr | Heterozygous | 1 |  | Cleft lip with or without cleft palate |
|  | Missense | c.71G>A | p.Gly24Glu | Heterozygous | 1 |  | Cleft lip with or without cleft palate |
|  | Missense | c.1159A>C | p.Met387Leu | Heterozygous | 8 |  | Cleft lip with or without cleft palate |
|  | Missense | c.1231G>C | p.Gly411Arg | Heterozygous | 8 |  | Cleft lip with or without cleft palate |
|  | Missense | c.1234G>A | p.Gly412Ser | Heterozygous | 8 |  | Cleft lip with or without cleft palate |
|  | Missense | c.1396G>A | p.Gly466Ser | Heterozygous | 8 |  | Cleft lip with or without cleft palate |
| Butali et al. 2014 | Missense | c.1282G>A | p.Asp428Asn | Heterozygous | 8 |  | Cleft lip |
|  | Missense | c.1396G>A | p.Gly466Ser | Heterozygous | 8 |  | Cleft lip with or without cleft palate |
|  | Splicing | c.952+2T>A | / | Heterozygous | / |  | Cleft lip |
| Leslie et al. 2015 | Missense | c.776C>T | p.Ala259Val | Heterozygous | 5 | Homeobox | Cleft lip with or without cleft palate |
| Gowans et al. 2017 | Missense | c.1223C>T | p.Pro408Leu | Compound heterozygous | 8 |  | Orofacial clefting |
| Monies et al. 2017 | Nonsense | c.433C>T | p.Arg145Term | Heterozygous | 3 | Paired box | Hypotonia, exercise intolerance, muscle weakness, creatine phosphokinase abnormalities |
| Proskorovski-Ohayon et al. 2017 | Splicing | c.1403-2A>G (NM_001135254.1) | / | Homozygous | / |  | Failure to thrive, severe global developmental delay, microcephaly, axial hypotonia, pyramidal signs, dystonic postures, seizures, irritability, and self-mutilation |
| Feichtinger et al. 2019 | Nonsense | c.220C>T | p.Arg74Term | Homozygous | 2 | Paired box | MYOSCO, postnatal growth retardation |
|  | Missense | c.166C>T | p.Arg56Cys | Homozygous | 2 | Paired box | MYOSCO, triangular face, postnatal growth retardation |
|  | Nonsense | c.433C>T | p.Arg145Term | Homozygous | 3 | Paired box | MYOSCO, micrognathia, postnatal growth retardation, arterial hypertension, renal atrophy and hydronephrosis |
|  | Splicing | c.86-1G>A | / | Homozygous | / |  | MYOSCO, hypotonic facies, high palate, postnatal growth retardation |
| Gaczkowaska et al. 2019 | Splicing | c.87G>A | / | Heterozygous | / |  | Cleft lip with or without cleft palate |
